# Management and risk factors of recurrent gestational trophoblastic neoplasia: An update from 2004 to 2017

**DOI:** 10.1002/cam4.2901

**Published:** 2020-02-05

**Authors:** Yujia Kong, Liju Zong, Hongyan Cheng, Fang Jiang, Xirun Wan, Fengzhi Feng, Tong Ren, Jun Zhao, Junjun Yang, Yang Xiang

**Affiliations:** ^1^ Department of Obstetrics and Gynecology Peking Union Medical College Hospital Chinese Academy of Medical Sciences and Peking Union Medical College Beijing China

**Keywords:** chemotherapy, gestational trophoblastic neoplasia, recurrence, surgery, survival rate

## Abstract

**Objective:**

We investigated the clinical characteristics, treatments, and survival of patients with gestational trophoblastic neoplasia (GTN) who experienced recurrence. Factors predictive of recurrence were also investigated.

**Methods:**

Patients with GTN who recurred after completing chemotherapy at Peking Union Medical College Hospital Trophoblastic Disease Center were identified between January 2004 and December 2017. Logistic regression analysis was used to identify factors predictive of GTN recurrence.

**Results:**

A total of 1827 patients with GTN achieved complete remission (CR) at our center, of whom 118 (6.5%) experienced recurrence during follow‐up. The recurrence rates for patients initially treated at our center and those referred to us were 2.7% and 14.6%, respectively. The majority of recurrent patients received floxuridine‐based multiagent chemotherapy (n = 64). Patients who underwent surgery achieved a significantly higher CR rate than those who did not (88.6% vs 61.1%, *P* = .001). Although 94.1% of recurrent patients reachieved CR, 33.3% of them recurred for a second time. The 5‐year survival rate of the entire cohort was 80.4%. An interval between antecedent pregnancy and chemotherapy >12 months (OR: 6.600, 95% CI [3.217‐13.540], *P* < .001), and an interval from first chemotherapy to achieving β‐human chorionic gonadotropin (β‐hCG) normalization >14 weeks (OR: 2.226, 95% CI [1.080‐4.588], *P* = .030) were predictors of recurrence.

**Conclusions:**

Patients with recurrent GTN are prone to recurring for a second time. Surgery plays a beneficial role in the management of recurrent GTN. An interval between antecedent pregnancy and chemotherapy >12 months, and an interval from first chemotherapy to achieving β‐hCG normalization >14 weeks were predictors of recurrence.

## Graphical abstract

The recurrence rate for patients with gestational trophoblastic neoplasia (GTN) was 6.5%, while the re‐recurrence rate for these recurrent patients increased to 33.3%. Surgery plays a beneficial role in the management of patients with recurrent GTN. An interval between antecedent pregnancy and chemotherapy >12 months, and an interval from first chemotherapy to achieving β‐human chorionic gonadotropin normalization >14 weeks were predictors of recurrence.

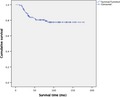

## 1 INTRODUCTION

Gestational trophoblastic neoplasia (GTN) encompasses a group of pregnancy‐related malignant tumors arising from abnormal placentas. GTN include invasive mole, choriocarcinoma, placental site trophoblastic tumor, and epithelioid trophoblastic tumor.^1^ Low‐risk GTN (International Federation of Gynecology and Obstetrics [FIGO] stage I‐III: score < 7) is treated with single‐agent chemotherapy and the overall survival rate approaches 100%. High‐risk GTN (FIGO stage II‐III: score ≥ 7 and stage IV) requires multiagent chemotherapy and achieves a survival rate of approximately 90%.^2^, ^3^ Although the majority of patients with GTN are cured with chemotherapy owing to the chemosensitivity of this tumor, a fraction of patients with GTN will experience recurrence after completion of treatment owing to chemoresistant disease. Two different investigations of patients with GTN who were treated at trophoblastic disease centers revealed recurrence rates of 3.5% and 4.7%.^4^, ^5^ Previous studies showed that patients with recurrent GTN had better outcomes than those with primary chemoresistant disease^6^, ^7^; the survival rates of patients with recurrent GTN were 77.8% and 93% in two previous studies.^6^, ^8^ However, the chemotherapy regimen that is most effective for patients with recurrent GTN remains unclear. Various salvage chemotherapy regimens are used worldwide, including etoposide, methotrexate, actinomycin‐D/etoposide, cisplatin (EMA/EP); paclitaxel, cisplatin/paclitaxel, etoposide (TP/TE); floxuridine, actinomycin‐D, vincristine (FAV); floxuridine, actinomycin‐D, etoposide, and vincristine (FAEV); bleomycin, etoposide, and cisplatin (BEP); etoposide, ifosfamide, and cisplatin or carboplatin (VIP or ICE); high‐dose chemotherapy with autologous bone marrow or stem cell transplantation; and immunotherapy.^2^, ^9^, ^10^ Owing to the rarity and the low recurrence rate of this disease, the previous studies of recurrent GTN contained small sample sizes, and limited information is available about the management and risk factors of recurrent GTN.

Therefore, we performed this retrospective study regarding recurrent GTN at the largest trophoblastic disease center in China. This study aims to analyze the characteristics, salvage treatments, and survival of patients with GTN who recurred after completion of treatment at our center; we also investigated factors predictive of GTN recurrence.

## 2 PATIENTS AND METHODS

### 2.1 Patients

The Peking Union Medical College Hospital (PUMCH) Gestational Trophoblastic Disease Center electronic database was screened to identify all patients with GTN treated at our center from January 2004 to December 2017. Complete remission (CR) was defined as consecutive normalization of serum β‐human chorionic gonadotropin (β‐hCG) levels for at least 4 weeks after chemotherapy. Recurrence was defined as increasing serum β‐hCG levels in the absence of a normal pregnancy 1 month after CR.

**Figure 1:**
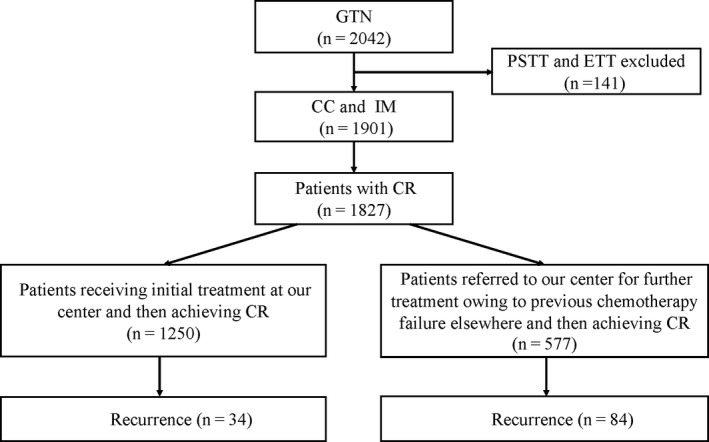
Flowchart of the patient selection process. CC, choriocarcinoma; CR, complete remission; ETT, epithelioid trophoblastic tumor; GTN, gestational trophoblastic neoplasia; IM, invasive mole; PSTT, placental site trophoblastic tumor

The patient selection process is shown in Figure 1. A total of 2042 patients with GTN were treated at our center from January 2004 to December 2017. Patients with placental site trophoblastic tumors and epithelioid trophoblastic tumors were excluded because of their variable chemosensitivity (n = 141). Among the 1901 patients with choriocarcinoma or invasive mole, 1827 achieved CR after completion of treatment at our center. As the largest GTN treatment center in China, patients with previous chemotherapy failure at other hospitals are often referred to our facility. Therefore, our patients were classified into two groups: those who received initial treatment at our center (n = 1250) and those referred to us for additional treatment due to previous chemotherapy failure elsewhere (n = 577). Subsequently, a total of 118 (6.5%) patients experienced recurrence during follow‐up. The database and medical records were reviewed to extract the clinical parameters, treatments, and outcomes at both initial presentation and recurrence. This retrospective study was performed with the approval of the ethics committee of PUMCH. Written informed consent was obtained from all study participants.

### 2.2 Treatment protocol

A number of factors should be considered when planning treatment for recurrent GTN, most notably the prerecurrence chemotherapy and the site of the recurrent tumor. Several salvage multiagent chemotherapy regimens were used at our center; the most common regimen was FAEV, the details of which were described previously.^11^ FAEV was administered to patients who had good responses to floxuridine‐based multiagent chemotherapies at initial treatment or to patients who had not previously been treated with this chemotherapy regimen. Furthermore, etoposide, methotrexate, and actinomycin‐D/cyclophosphamide and vincristine (EMA/CO) was administered to patients who were resistant to floxuridine‐based multiagent chemotherapy at initial treatment. Patients who remained refractory were considered eligible for platinum‐based chemotherapy (EMA/EP and TP/TE).

Imaging examinations were performed at the time of recurrence. Magnetic resonance imaging (MRI) of pelvis and plain computed tomography (CT) of chest was performed to evaluate the presence of uterine disease and metastases to lung. Contrast enhancement CT of abdomen and MRI of brain were also considered for patients with suspected metastases to abdominal organs and brain. Indications of salvage surgery in patients with recurrent GTN were as follows: (a) the patient's general condition should be well enough to tolerate surgery, (b) there should be no evidence of active disease outside the site of resection, (c) the foci of chemoresistant disease should be localized and resectable, and (d) preoperative serum β‐hCG levels should be as low as possible, ideally less than 10 IU/L.^12^, ^13^ Moreover, combined surgeries were performed in patients with resectable metastases in multiple sites, including hysterectomy ± pulmonary lobectomy ± craniotomy.

### 2.3 Evaluation

Serum β‐hCG levels were measured weekly to evaluate the response to chemotherapy. After completion of chemotherapy, serum β‐hCG levels were monitored weekly for 4 weeks, then monthly for 1 year, followed by 6 months for 3 years, and then once a year till 5 years, then once every 2 years afterwards. Resistance referred to plateaued or elevated β‐hCG levels after two to three courses of chemotherapy.

### 2.4 Statistical analysis

Categorical variables were compared using Pearson's chi‐square test or Fisher's exact test. Continuous variables were compared using Wilcoxon‐Mann‐Whitney *U* test for nonnormally distributed variables. The survival time was measured from the date of diagnosis to the date of last follow‐up or death; the overall survival rates were estimated using the Kaplan‐Meier method and compared using log‐rank tests. Univariate analysis was performed with Pearson's chi‐square test or Fisher's exact test to reveal possible risk factors of recurrence. Subsequently, factors revealed in the univariate analysis were brought into multivariate analysis with logistic regression to identify factors predictive of recurrence. All the analyses were performed using the Statistical Package for Social Sciences (SPSS version 23.0; IBM Corp.). *P* < .05 were considered statistically significant.

## 3 RESULTS

### 3.1 Patients characteristics at initial presentation for recurrent GTN

A total of 1827 patients with GTN achieved CR at our center, including 1250 patients who received initial treatment at our center and 577 patients who were referred to us. Among the 1827 patients, 118 (6.5%) experienced recurrence during follow‐up. Of 118 patients with recurrence, 34 patients received initial treatment at our center and recurred after CR, while 84 failed chemotherapy elsewhere and were referred to us for further treatment, after which they achieved CR but then recurred. These two groups are incomparable, because patients referred to us received more lines of chemotherapy than patients initially treated at our center. Thus, we only present these patients’ characteristics separately in two groups without any comparison in Table 1. At initial presentation, 26 patients with low‐risk GTN were treated with actinomycin‐D or floxuridine‐based multiagent chemotherapy, while 92 patients with high‐risk GTN were treated with multiagent chemotherapy (including FAV, FAEV, and EMA/CO). Overall, 118 patients in our study had received a median of 6 courses of chemotherapy at initial treatment, including three courses of consolidation chemotherapy. Forty‐three patients had undergone surgeries prerecurrence.

**Table 1:** Characteristics of patients with recurrent GTN at initial presentation at our center (n = 118)

Characteristics	Total cohort (n = 118)	Recurrent GTNs initially treated at our center (n = 34)	Recurrent GTNs referred to our center (n = 84)
Age (yr, median)	31	31	31
Antecedent pregnancy			
Mole	42	11	31
Abortion	47	15	32
Term	29	8	21
Interval from index pregnancy to initial chemotherapy (mo)			
≤12	34	15	19
>12	84	19	65
Pretreatment serum β‐hCG (IU/L)			
<10^3^	58	10	48
10^3^‐10^4^	28	6	22
10^4^‐10^5^	26	13	13
>10^5^	6	5	1
Diagnosis			
Invasive mole^a^	17	9	8
Choriocarcinoma^a^	101	25	76
Stage			
Ⅰ	25	9	16
Ⅱ	1	0	1
Ⅲ	76	21	55
Ⅳ	16	4	12
FIGO score			
0‐6 (low‐risk)	26	16	10
7‐12 (high‐risk)	77	16	61
≥13 (ultra high‐risk)	15	2	13
Total chemotherapy courses (median)	6	7	6
Chemotherapy to achieve β‐hCG normalization (courses, median)	3	4	3
Consolidation chemotherapy (courses, median)	3	3	3
Surgery			
Yes	43	11	32
No	75	23	52

Abbreviations: β‐hCG, β‐human chorionic gonadotropin; FIGO, International Federation of Gynecology and Obstetrics; GTN, gestational trophoblastic neoplasia.

a The initial diagnosis of these patients refers to clinical diagnosis based on antecedent pregnancy and interval from index pregnancy to chemotherapy, while 43 of these patients had pathological evidence.

### 3.2 Details of GTN recurrence

The recurrence rates for patients initially treated at our center and those referred to us were 2.7% and 14.6%, respectively. Specifically, among the patients initially treated at our center, the recurrence rates for patients with low‐ and high‐risk disease were 1.6% and 6.9%. Similarly, the low‐ and high‐risk recurrence rates for patients referred to our center were 5.0% and 19.6%, respectively (Table 2).

**Table 2:** Details of recurrence rates in patients with GTN (n = 1827)

	Patients initially treated at our center	Patients referred to our center
Total cohort		
CR	1250	577
Recurrence	34	84
Recurrence rate (%)	2.7	14.6
Re‐recurrence rate (%)	27.3 (9/33)	35.9 (28/78)
Low‐risk disease at initial presentation		
CR	988	200
Recurrence	16	10
Recurrence rate (%)	1.6	5.0
Re‐recurrence rate (%)	13.3 (2/15)	10.0 (1/10)
High‐risk disease at initial presentation		
CR	262	377
Recurrence	18	74
Recurrence rate (%)	6.9	19.6
Re‐recurrence rate (%)	38.9 (7/18)	39.7 (27/68)

Abbreviations: CR, complete remission; GTN, gestational trophoblastic neoplasia.

The median time to recurrence was 3 months (range = 1‐84 months); the majority of recurrences (78.8%) occurred within 1 year of completion of treatment and only two patients recurred after 5 years of follow‐up (Table 3). A nonmolar antecedent pregnancy (*P* < .001) and high‐risk GTN at initial presentation (*P* = .017) significantly increased the risk of a recurrence within 3 months of completing chemotherapy (Table 4).

**Table 3:** Time to recurrence after completing initial chemotherapy at our center (n = 118)

Time to recurrence (mo)	Total cohort		Recurrent GTNs initially treated at our center		Recurrent GTNs referred to our center	
	No.	Cumulative (%)	No.	Cumulative (%)	No.	Cumulative (%)
0‐3	61	51.7	17	50.0	44	52.4
4‐6	18	66.9	4	61.8	14	69.0
7‐12	14	78.8	6	79.4	8	78.6
13‐24	14	90.7	3	88.2	11	91.7
25‐60	9	98.3	3	97.0	6	98.8
>60	2	100	1	100	1	100

Abbreviation: GTN, gestational trophoblastic neoplasia.

**Table 4:** Association between clinical variables and time to recurrence (n = 118)

	Time to recurrence ≤ 3 mo (n = 61)	Time to recurrence> 3 mo (n = 57)	*P*
Age (yr, median)	32	29	0.094
Antecedent pregnancy			
Mole	13	29	<0.001*
Abortion or term	48	28	
Interval from index pregnancy to initial chemotherapy (mo)			
≤12	13	21	0.063
>12	48	36	
Stage			
Ⅰ	11	14	0.706
Ⅱ	1	0	
Ⅲ	41	35	
Ⅳ	8	8	
FIGO score at initial presentation			
0‐6 (low‐risk)	7	19^a^	0.017*
7‐12 (high‐risk)	45	32^b^	
≥13 (ultra high‐risk)	9	6a,b	
Previous chemotherapy failure			
Yes	44	40	0.815
No	17	17	
Consolidation chemotherapy (courses, median)	3	3	0.097

Abbreviations: FIGO, International Federation of Gynecology and Obstetrics; GTN, gestational trophoblastic neoplasia.

^a,b^ A subset of FIGO score categories whose recurrence rate differs significantly from each other at the *P* < .05 level. If the two subgroups are marked with different subscript letter, there is statistically significant difference between the two subgroups. Otherwise, there is no statistically significant difference between the two subgroups.

* *P* < .05 indicated statistically significant differences.

### 3.3 Treatment for patients with recurrent GTN

The majority of recurrent patients (n = 64) received FAEV as the preferred chemotherapy regimen. Among these 64 patients, 55 (85.9%) achieved CR and 9 (14.1%) switched to EMA/CO or EMA/EP owing to their unresponsiveness to FAEV (n = 7) or toxicity (n = 2). EMA/CO was also administered to the other 40 patients who failed to floxuridine‐based multiagent chemotherapy at initial treatment. Among the 40 patients treated with EMA/CO, 32 (80.0%) achieved CR while 8 (20.0%) switched to platinum‐based chemotherapy (including EMA/EP and TE/TP). Additionally, platinum‐based chemotherapy was also administered to the remaining 14 patients and 11 of them achieved CR.

In addition to chemotherapy, salvage surgery was performed in 81 (68.6%) patients who recurred, among whom 30 patients underwent hysterectomy or uterine lesions resection; 34 underwent pulmonary lobectomy; 9 underwent hysterectomy combined with pulmonary lobectomy; 3 underwent craniotomy plus hysterectomy; 3 underwent craniotomy; 1 underwent adnexectomy; and 1 underwent tumor resection in the adrenal gland. Overall, patients who underwent salvage surgeries achieved a significantly higher CR rate than those who did not undergo such surgeries (88.6% vs 61.1%, *P* = .001).

Furthermore, five patients with brain metastases at recurrence underwent stereotactic radiotherapy after craniotomy, three of whom achieved CR. Three patients with unresectable lung metastases received thoracic radiotherapy; all ultimately achieved CR. Four patients who experienced repeated recurrences were treated with anti‐PD‐1 immunotherapy (pembrolizumab), of whom two had good responses and have remained in remission more than 4 months later.

### 3.4 Outcomes of patients with recurrent GTN

**Figure 2:**
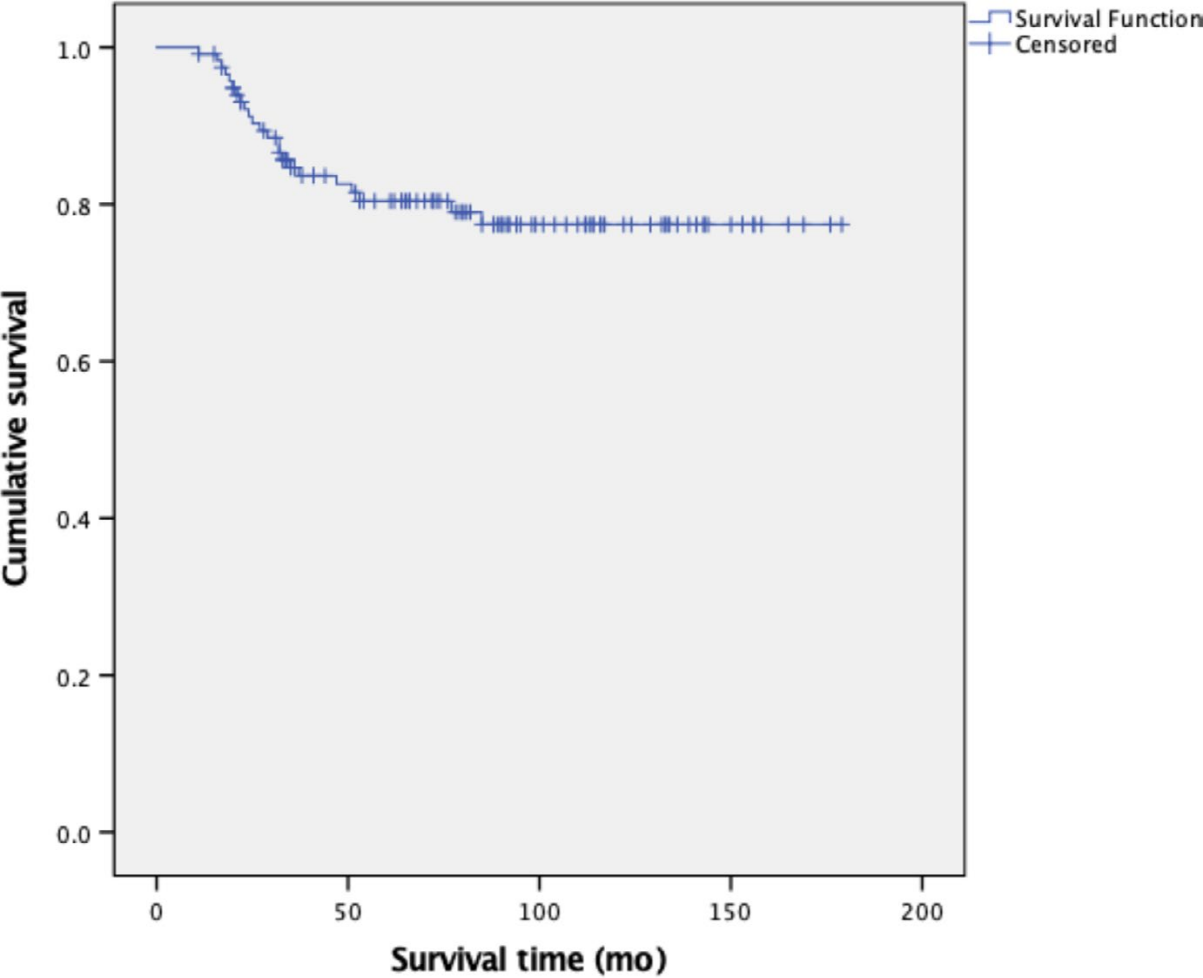
Kaplan‐Meier curve showing the overall survival of 118 patients with recurrent gestational trophoblastic neoplasia

One hundred and eleven of the 118 patients (94.1%) achieved CR again after completing salvage treatment, while the remaining 7 (5.9%) experienced disease progression owing to resistance to chemotherapy. After a median follow‐up of 76 months (range = 11‐179 months), 37 patients recurred for a second time; as such, the re‐recurrence rate was 33.3% (Table 2). The median interval between the first and second recurrence was 3 months (range = 1‐48 months). Of the 37 re‐recurred patients, only 25 (67.6%) re‐achieved CR and the remaining 12 (32.4%) patients experienced disease progression. Ultimately, 11 of 25 patients experienced three to four times recurrences. Overall, 23 patients (19.5%) died of progressive disease, among whom 7 and 16 died during their first and subsequent recurrences, respectively. At the last follow‐up, 92 patients were alive and 3 were lost to follow‐up. The estimated 5‐year overall survival rate of patients with recurrent GTN was 80.4% (Figure 2). There was no statistically significant difference between the survival rates of the recurrent patients who were initially treated at our center and those who were referred (89.6% vs 76.8%, *P* = .159).

### 3.5 Predictors of GTN recurrence

There is great heterogeneity in patients referred to us, because they have received several different prior chemotherapy regimens elsewhere. Therefore, we only explore the factors predictive of GTN recurrence among the 1250 patients who received initial treatment at our center. Patients with high‐risk GTN had a significant higher recurrence rate than patients with low‐risk disease (6.8% vs 1.6%, *P* < .001), while there was no statistically significant difference between patients with ultra high‐risk GTN and the other two subgroups (Table 5). Nonmolar antecedent pregnancy (*P* < .001), an interval between antecedent pregnancy and chemotherapy >12 months (*P* < .001) and an interval from first chemotherapy to achieving β‐hCG normalization >14 weeks (*P* = .001) were also found to have significant correlations with recurrence in the univariate analysis (Table 5). Multivariate analysis with logistic regression revealed that an interval between antecedent pregnancy and chemotherapy >12 months (OR: 6.600, 95% CI [3.217‐13.540], *P* < .001), and an interval from first chemotherapy to achieving β‐hCG normalization >14 weeks (OR: 2.226, 95% CI [1.080‐4.588], *P* = .030) were predictors of recurrence among patients with GTN (Table 5).

**Table 5:** Univariate and multivariate analysis of risk factors that predict GTN recurrence for patients initially treated at our center (n = 1250)

	Univariate		Multivariate	
	Recurrence rate (%)	*P*	OR (95% CI)	*P*
Age (yr)				
<40	2.6 (n = 27)	0.495	–	–
≥40	3.4 (n = 7)		–	–
Antecedent pregnancy				
Mole	1.3 (n = 11)	<0.001*	–	–
Abortion or term	5.3 (n = 23)		–	–
Interval from index pregnancy to initial chemotherapy (mo)				
≤12	1.4 (n = 15)	<0.001*	1	–
>12	10.3 (n = 19)		6.600 (3.217‐13.540)	<0.001*
Stage				
Ⅰ‐Ⅲ	2.5 (n = 30)	0.055	–	–
Ⅳ	7.4 (n = 4)		–	–
FIGO score				
0‐6 (low‐risk)	1.6 (n = 16)^a^	<0.001*	–	–
7‐12 (high‐risk)	6.8 (n = 16)^b^		–	–
≥13 (ultra high‐risk)	7.4 (n = 2)a,b		–	–
Interval between first chemotherapy and β‐hCG normalization (wk)				
≤14 wk	1.7 (n = 15)	0.001*	1	–
>14 wk	4.8 (n = 19)		2.226 (1.080‐4.588)	0.030*

Abbreviations: β‐hCG, β‐human chorionic gonadotropin; FIGO, International Federation of Gynecology and Obstetrics; GTN, gestational trophoblastic neoplasia.

^a,b^ A subset of FIGO score categories whose recurrence rate differed significantly from each other at the *P* < .05 level. If the two subgroups were marked with different subscript letter, there was statistically significant difference between them. Otherwise, there was no statistically significant difference between the two subgroups.

* *P* < .05 indicated statistically significant differences.

## 4 DISCUSSION

Although the majority of patients with GTN can be cured by chemotherapy, a minority experience recurrence after CR because of chemoresistant disease. Previous studies of recurrent GTN included small sample sizes owing to this condition's rarity and limited information is available; as such, our study included a large series of patients with recurrent GTN to investigate the management and survival of these patients. Moreover, we revealed risk factors for recurrence in GTN. Our previous cohort study revealed a recurrence rate of 3.4% (n = 31) among 1130 patients with GTN who were treated at our center between January 1985 and January 2004^14^; our current study's cohort (that was treated at our center between January 2004 and December 2017) had a higher recurrence rate of 6.5%. The trend of increased recurrence rate might attribute to more referred patients who failed chemotherapy at local hospitals in the past decade. To minimize the heterogeneity in our cohort, we analyzed the data separately in these two groups: those who received initial treatment at our center and those referred to us. It appears that patients referred to us showed a higher recurrence rate than patients initially treated at our center (14.6% vs 2.7%). However, statistical evaluation of comparison between the two groups was not performed, because patients referred to us had received more lines of chemotherapy than patients initially treated at our center so that the two groups were incomparable. What is clear is that the recurrence rate for patients initially treated at our center is quite low, which is slight lower than that of our previous cohort (2.7% vs 3.6%).^14^ The greater likelihood of recurrence among referred patients may be a consequence of prior chemotherapy resistance at other hospitals.

Although the majority of recurrent patients (94.1%) re‐achieved CR after salvage treatment, the re‐recurrence rate for these patients increased to 33.3%; moreover, the CR rate among patients who experienced a second recurrence was only 67.6%. This indicated that recurrent patients with GTN were likely to re‐experience such recurrence, and that such patients became refractory. Ultimately, the 5‐year survival rate of recurrent patients in our cohort was 80.4%, which was lower than that of patients who were initially diagnosed.^2^ Furthermore, subgroup analysis showed that the 5‐year survival rate for recurrent patients initially treated at our center was 89.6%, which was close to that at the Charing Cross Trophoblastic Disease Center.^6^ However, the recurrent patients referred to us achieved a much lower survival rate of 76.8%, although the difference was not statistically significant. Prior chemotherapy resistance before their referral might not only cause the greater likelihood of recurrence, but also ultimately leads to the poorer prognoses of these patients.

Our study revealed that the median time to recurrence among patients with GTN was 3 months; the majority of these patients (78%) recurred within 1 year after the completion of treatment. These findings are very similar to those recently reported from the UK,^5^ which illustrated the importance of intensive monitoring of serum β‐hCG for at least an additional year after achieving CR. Notably, nearly 10% of patients recurred after 2 years, which also emphasizes the necessity of long‐term monitoring of serum β‐hCG for such patients. Moreover, a nonmolar antecedent pregnancy and high‐risk disease at initial presentation might increase the risk of a recurrence within 3 months of completing chemotherapy.

Several salvage chemotherapy regimens are used worldwide for patients with GTN who are chemoresistant or experience recurrence. It has been reported that EMA/EP was an effective salvage regimen for patients who were resistant to EMA/CO, producing cure rates ranging from 66.6% to 84.9%.^15^, ^16^, ^17^ Wang et al demonstrated that TP/TE produced a cure rate that was comparable to that of EMA/EP, but with less toxicity.^18^ At our center, however, the FAEV regimen was used not only as a first‐line chemotherapy for patients with high‐risk GTN, but also as salvage treatment for patients who had good responses to floxuridine‐based multidrug chemotherapy at initial treatment or those who had not previously been treated with this regimen. Yang et al reported that 80% of their patients with stage IV GTN who received FAEV as primary treatment achieved CR.^19^ Furthermore, Feng et al also showed that the FAEV regimen produced a CR rate of 60.4% in patients with chemoresistant or recurrent GTN.^20^ In our current study, 85.9% of patients with recurrent GTN who were treated with the FAEV regimen achieved CR, which demonstrates the efficiency of this chemotherapy regimen for such patients.

Surgery also plays an important role in the management of patients with recurrent GTN. Previous studies have shown that salvage surgery is an effective adjunct curative treatment for patients with GTN who are resistant to chemotherapy.^13^, ^21^, ^22^, ^23^, ^24^, ^25^, ^26^, ^27^ As most GTN recurrences are caused by chemoresistant disease, resection of the chemoresistant tumor in the uterus, lung, liver, or other organs should be considered upon GTN recurrence. In our series, 68% of recurrent patients underwent salvage surgery combined with chemotherapy; these patients achieved a much higher CR rate than those who did not undergo salvage surgeries. This finding also emphasizes the importance of salvage surgery in terms of removing the foci of persistent or chemoresistant disease in patients with recurrent GTN.

The majority of GTNs are curable; however, since a minority of patients experience recurrence, it is important to identify the factors that predict such recurrences. It was inappropriate to assess risk factors for recurrence in the entire cohort due to the heterogeneity in patients who were referred to us. Therefore, we only explored the factors predictive of GTN recurrence among the patients who were initially treated at our center. Patients with high‐risk GTN had a significant higher recurrence rate than patients with low‐risk disease, while there was no statistically significant difference between patients with ultra high‐risk GTN and the other two subgroups (low and high risk), which might attribute to the small number of ultra high‐risk GTN in our cohort. Our multivariate analysis revealed that an interval between antecedent pregnancy and chemotherapy >12 months, and an interval from first chemotherapy to achieving β‐hCG normalization >14 weeks were both predictors of GTN recurrence. Patients with these unfavorable factors should be monitored more carefully during follow‐up to detect any recurrences at early stage.

Given the rarity and the low recurrence rate of this disease, we were only able to conduct a retrospective study of recurrent GTN. Thus, our study had limitations inherent to retrospective study designs. Another limitation is that our cohort consisted of patients initially treated at our center and patients referred to us, which is a heterogeneous population. However, to minimize the heterogeneity in this study, we further analyzed the data separately in two groups. In addition, as the largest trophoblastic disease center in China, our cohort contained a very high proportion of high‐risk patients due to the referrals from local hospitals, which might not reflect general experience of GTN. However, this study provided valuable experience on this disease from a specialized center.

In conclusion, the recurrence rate for patients with GTN was quite low. Although the majority of recurrent patients re‐achieved CR after salvage treatment, they were prone to recurring again. Several salvage chemotherapy regimens can be used to treat recurrent GTN, and the selection of chemotherapy regimens is dependent on previous chemotherapy. Surgery plays a beneficial role in the management of recurrent GTN. The majority of patients with recurrent GTN achieved favorable outcomes. An interval between antecedent pregnancy and chemotherapy >12 months, and an interval from first chemotherapy to achieving β‐hCG normalization >14 weeks were predictors of GTN recurrence.

## CONFLICT OF INTEREST

The authors have no conflict of interest to declare.

## AUTHOR CONTRIBUTIONS

YJ Kong, JJ Yang, and Y Xiang contributed to study design and conception. HY Cheng, F Jiang, FZ Feng, and XR Wan contributed to acquisition of data. YJ Kong, T Ren, and J Zhao contributed to analysis and interpretation of data. YJ Kong, LJ Zong, and JJ Yang contributed to drafting the manuscript. All authors reviewed and approved the final manuscript.

## Data Availability

The raw data supporting the conclusions of this manuscript will be made available by the authors, without undue reservation, to any qualified researcher.
